# A therapy-grade protocol for differentiation of pluripotent stem cells into mesenchymal stem cells using platelet lysate as supplement

**DOI:** 10.1186/scrt540

**Published:** 2015-01-12

**Authors:** Carlos Luzzani, Gabriel Neiman, Ximena Garate, María Questa, Claudia Solari, Darío Fernandez Espinosa, Marcela García, Ana Lía Errecalde, Alejandra Guberman, María Elida Scassa, Gustavo Emilio Sevlever, Leonardo Romorini, Santiago Gabriel Miriuka

**Affiliations:** Laboratorio de Biología del Desarrollo Celular, LIAN-Unidad Asociada al CONICET, Fundación FLENI, Ruta 9, Km53, Belen de Escobar, Argentina; Laboratorio de Regulación de Expresión Génica, Facultad de Ciencias Exactas y Naturales, Universidad de Buenos Aires, Intendente Güiraldes 2160, Buenos Aires, Argentina; Cátedra de Citología, Histología y Embriología, Facultad de Ciencias Médicas, Universidad Nacional de La Plata, Calle 60 s/n, 1900 La Plata, Argentina; Investigador, Consejo Nacional de Investigaciones Científicas y Técnicas (CONICET), Buenos Aires, Argentina

## Abstract

**Introduction:**

Mesenchymal stem cells (MSCs) are a promising source of cells for regenerative therapies. Although they can be isolated easily from several tissues, cell expansion is limited since their properties are lost with successive passages. Hence, pluripotent derived MSCs (PD-MSCs) arise as a suitable alternative for MSC production. Nevertheless, at present, PD-MSC derivation protocols are either expensive or not suitable for clinical purposes.

**Methods:**

In this work we present a therapy-grade, inexpensive and simple protocol to derive MSCs from pluripotent stem cells (PSCs) based on the use of platelet lysate (PL) as medium supplement.

**Results:**

We showed that the PD-MSC_PL_ expressed multiple MSC markers, including CD90, CD73, CD105, CD166, and CD271, among others. These cells also show multilineage differentiation ability and immunomodulatory effects on pre-stimulated lymphocytes. Thorough characterization of these cells showed that a PD-MSC_PL_ resembles an umbilical cord (UC) MSC and differs from a PSC in surface marker and extracellular matrix proteins and integrin expression. Moreover, the OCT-4 promoter is re-methylated with mesenchymal differentiation comparable with the methylation levels of UC-MSCs and fibroblasts. Lastly, the use of PL-supplemented medium generates significantly more MSCs than the use of fetal bovine serum.

**Conclusions:**

This protocol can be used to generate a large amount of PD-MSCs with low cost and is compatible with clinical therapies.

**Electronic supplementary material:**

The online version of this article (doi:10.1186/scrt540) contains supplementary material, which is available to authorized users.

## Introduction

Mesenchymal stem cells (MSC), sometimes also addressed as mesenchymal stromal cells, have been isolated from many different tissues – and although some differences may be found according to their origin, most of them share their main features, including multipotent differentiation and immunomodulation [1]. Irrespective of the source of isolation, MSC have been found to be able to modulate the immune response. This feature has been extensively studied *in vitro* and *in vivo* in the past years, and MSC are currently assessed in clinical trials for their efficacy in the treatment of many immune-related diseases. Although MSC can be easily isolated from tissues such as bone marrow, umbilical cord or adipose tissue, it has been reported that these cells lose their properties rapidly with time, undergoing cellular senescence [2, 3]. Moreover, it is possible that some therapies will require large and repeated doses of MSC. In the case that these therapies involve autologous MSC, there would be some limitations in the number of repeated procedures to obtain the cells. A limitless, economic source of MSC would therefore be a valid alternative when thinking in an autologous, off-the-shelf MSC therapy.

Platelet lysate (PL) is increasingly used instead of fetal bovine serum (FBS) as a medium supplement for growing MSC. PL’s advantages have been described extensively, and include its biocompatibility with cell therapy, low cost, and easiness to produce [4, 5]. PL contains a very significant amount of growth factors, released by the platelets after lysing in the freeze/thaw cycles [6–8]. These growth factors are involved in many relevant functions in stem cell biology, including basic fibroblast growth factor, insulin-like growth factor and transforming growth factor beta. Moreover, it has been demonstrated that growing MSC in PL-supplemented medium preserves the immunomodulatory ability of the cells [9]. PL supplement has been already used to grow MSC with success, and these cells are used in clinical trials involving MSC without presenting any adverse reaction [10].

Pluripotent stem cells (PSC) can differentiate into any type of adult stem cell. Interestingly, it has been reported that PSC can derive into cells that share many features with MSC isolated from adult tissues, and hence they have been called pluripotent-derived mesenchymal stem cells (PD-MSC) [11–13]. Many papers have described different protocols to derive PD-MSC, and some of them involve some complex manipulations or the use of cell separation methods [14–22]. Even though they are called mesenchymal cells, there are some disagreements between some papers regarding the identity of PD-MSC, and some authors consider that these cells are not related to MSC, based on their gene expression profile [23]. In any case, PD-MSC have been analyzed in many reports and they share many of the features of the adult MSC, including surface markers, multilineage differentiation and immunomodulation. Finally, there are some reports that have analyzed their therapeutic potential, and these cells have been shown to be very potent immunomodulators in animal models [24–27].

We have developed a method to derive PD-MSC using PL as a media supplement (PD-MSC_PL_). This protocol generates a very significant number of PD-MSC within 3 to 4 weeks in a robust and consistent way. We believe that this method can be scaled up at low cost to produce a significant number of PD-MSC_PL_ useful for clinical therapies.

## Materials and methods

### Cells and cell pluripotent stem cell culture methods

H9 human embryonic stem cells (hES) were purchased from WiCell (Madison, Wisconsin, USA). Induced pluripotent stem cells (iPS) were generated in our laboratory (Maria Questa et al., unpublished observations) by standard techniques. Briefly, foreskin fibroblasts were reprogrammed by transfection with the STEMCCA lentivirus vector, generously obtained from Gustavo Mostovslasky [28]. Several clones have been characterized in our laboratory by demonstrating their pluripotent state and its ability to differentiate into cells from the three germinal layers and by the formation of teratomas. For this paper, we have used both clones FN2.1 and FH. PSC, either hES or iPS, are regularly grown in our laboratory over inactivated (by radiation) mouse embryonic fibroblasts, in Dulbecco’s modified Eagle’s medium (DMEM)/F12 medium supplemented with 10% Knock-Out Serum Replacement, 8 ng/ml basic fibroblast growth factor, and penicillin–streptomycin (all from Life Technologies, Carlsbad, California, USA), under standard culture conditions (37°C with a 5% carbon dioxide humidified atmosphere). Medium is changed daily. Human umbilical cord mesenchymal stem cells (UC-MSC) were isolated from Wharton jelly tissues. Derivation of UC-MSC was done with due consent from the donor’s parents. Small pieces of the umbilical cord, excluding the major vessels, were layered onto plastic and cultured in alpha modified Eagle’s medium supplemented with 10% PL and penicillin–streptomycin. Fibroblasts were obtained from the foreskin of a patient undergoing scheduled surgery, with informed consent from the parents. These cells are grown in DMEM supplemented with 10% FBS and antibiotics. All isolations of primary cells were approved by FLENI Ethical Committee after reviewing the research protocol.

### Platelet lysate preparation

PL was produced with some modifications as described previously [5]. Expired platelet bags were obtained from the Hemotherapy Service of FLENI Foundation and frozen at -80°C for at least 24 hours. The units were then thawed at 37°C, pooled under sterile conditions, and frozen again at -80°C. We prepared several batches, each one containing at least 10 units of platelets obtained from at least five different patients. Finally, the pooled units were again thawed, aliquoted, and centrifuged at 3,000 × *g* for 30 minutes. The supernatant was stored at -80°C until use. To use as a supplement, aliquots were thawed and 2 IU/ml heparin (Sigma-Aldrich, San Luis, Missouri, USA) were added before medium supplementation. Alternatively, we modified the protocol described by Copland and colleagues for generating fibrinogen-depleted PL [29]. In this protocol, fibrinogen is excluded by adding 10 mM CaCl_2_ for 1 hour at 37°C. This step generates a dense clot; we then centrifuged the PL at 3,000 × *g* for 1 hour, collected the supernatant and froze it at -80°C. We found no difference with either method of PL generation regarding the success of PD-MSC differentiation. However, we prefer this later protocol since it avoids occasional gelatinization of the medium and produced a supplement with less debris without affecting results.

### Pluripotent-derived mesenchymal stem cell differentiation

On day 0 of differentiation, PSC grown either on inactivated mouse embryonic fibroblasts, Matrigel or Geltrex were incubated with Accutase (Life Technologies) until cells were completely dissociated, and were plated onto Matrigel-coated plates (diluted 1/40; BD, San Jose, California, USA) or onto Geltrex-coated plates (diluted 1/100; Life Technologies). Cells were resuspended in alpha modified Eagle’s medium supplemented with 10% PL, penicillin–streptomycin (Life Technologies) plus B27 diluted 1/100 (Life Technologies). A Rho kinase inhibitor (10 μM Y27632; Tocris, Avon, Bristol, UK) was added every time the cells were passed until day 14 of differentiation. From that day onward, cells were passed using trypsin on plastic dishes with no coating, and were grown in medium supplemented with 10% PL and penicillin–streptomycin. As an alternative method of differentiation of PD-MSC, we passed the cells in the same way but grew them in DMEM supplemented with 10% FBS (PAA, Pasching, Austria) plus penicillin–streptomycin. Characterization experiments were performed after 30 days of differentiation and up to three passages later, unless otherwise stated.

### Fluorescent microscopy

Adherent cells were fixed with 4% paraformaldehyde at room temperature for 45 minutes and then washed three times with phosphate-buffered saline (PBS) with 0.1% bovine serum albumin (BSA). Cells were then permeabilized in PBS/BSA, 10% normal goat serum and 0.1% Triton for 45 minutes, and then washed again three times. Samples were then incubated at 4°C overnight with either mouse anti-OCT-4 (1:200, clone C-10; Santa Cruz, Dallas, Texas, USA) or rabbit anti-NANOG (1:200, D73G4; Cell Signaling, Danvers, Massachusetts, USA) primary antibodies in PBS/BSA with 10% normal goat serum. After washing three times with PBS/BSA, cells were incubated for 45 minutes at room temperature with the corresponding secondary antibody (goat anti-mouse ALEXA-488, 1:100; Life Technologies) or goat anti-rabbit ALEXA-555 conjugated antibody (1:100; Life Technologies) plus 0.1 μg/ml 4′,6-diamidino-2-phenylindole. Cells were finally washed three times. Images were acquired using a Nikon Eclipse TE2000-S inverted microscope (Microlat, Buenos Aires, Argentina) and Eclipse Net software (Microlat, Buenos Aires, Argentina).

### Cell number quantification

PSC (1.5 × 10^5^ cells) were initially plated at day 0 of the protocol, on DMEM supplemented with either 10% PL or 10% FBS. Cells were grown as described in the protocol. Every time the cells were passed, a 100 μl aliquot was run through a BD Accuri cytometer and the cell number concentration was determined.

### Flow cytometry

Flow cytometry analyses were performed in a BD Accuri cytometer. A standard protocol for cell staining was used. Briefly, adherent cells were washed with PBS and a single-cell suspension was obtained by incubating them with Accutasse. Cells were then washed with PBS plus 0.5% albumin, and were incubated at room temperature for 30 minutes with the appropriate antibody concentration. Cells were then washed with PBS plus 0.5% albumin and analyzed. At least 5,000 events were counted. A list of the antibodies used in this paper is presented in Table S1 in Additional file 1.

### PCR and quantitative RT-PCR

Total RNA from cell extracts was isolated using Trizol (Life Technologies) as described by the manufacturer. Total RNA (1 μg) was retrotranscribed using MMLV (Promega, Madison, Wisconsin, USA) and random primers (Life Technologies). Quantitative RT-PCR was performed using the Sybr Green ER qPCR Super Mix (Life Technologies) in a Step One Plus Real-Time PCR system (Applied Biosystems, Foster City, California, USA). The list of the primers used is presented in Table S2 in Additional file 1.

### Multilineage differentiation of pluripotent-derived mesenchymal stem cells

Multilineage differentiation potential was assessed using the StemPro Adipogenesis, Osteogenesis or Chondrogenesis Kit (Life Technologies) as per the instructions of the manufacturer. After differentiation, adipogenesis induction was assessed by Sudan Black staining. Briefly, cells were fixed with 4% paraformaldehyde for 45 minutes at room temperature, stained with a Sudan Black saturated solution in 70% ethanol for 5 minutes at room temperature, and finally washed thoroughly with 70% ethanol. Osteogenesis induction was assessed by Alzarin Red staining. Fixed cells were stained in a 2% Alzarin Red solution (pH 4.2) for 3 minutes at room temperature and then washed thoroughly with distilled water. Finally, chondrogenic induction was assessed by Alcian Blue staining. Fixed cells were stained in a 1% Alcian Blue solution prepared in 0.1 N HCl for 30 minutes at room temperature and then washed thoroughly with distilled water. All three preparations were visualized under a light microscope. Images were acquired using a Nikon Eclipse TE2000-S inverted microscope and Eclipse Net software.

### Immunomodulation experiment

Peripheral blood mononuclear cells (PBMC) were isolated from whole blood of healthy donors. Briefly, heparinized blood samples were resuspended in PBS (1:1). Diluted blood was then carefully layered on top of the Ficoll (Histopaque-1077; Sigma, San Luis, Missouri, USA) at a ratio of 1.5:1 and centrifuged at 800 × *g* for 30 minutes. The buffy coat layer containing mononuclear cells was aspirated, and the PBMC were then either cultured for immediate use in RPMI-1640 (Gibco, Carlsbad, California, USA) supplemented with 10% FBS or frozen for later use. Before the experiment, an appropriate number of PBMC were stained with CFSE (Life Technologies) as described previously [30] and prestimulated with concanavalin-A (Sigma) for 24 hours. Then, 1-day stimulated PBMC were washed with PBS and cocultured with MSC for 96 hours in RPMI-1640 supplemented with 10% FBS, 10 ng/ml anti-CD3 antibody (clone UCHT1) and 300 IU/ml interleukin-2 (both from R&D Systems, Minneapolis, Minnesota, USA). PBMC where then collected and analyzed by flow cytometry. In some cases, we stained the PBMC for CD4 and CD8 antigens. Unstained PBMC, stained but not stimulated PBMC and stained stimulated PBMC without MSC were also run as controls.

### Promoter methylation analysis

Genomic DNA was isolated using the Wizard Genomic DNA kit (Promega). Then 200 ng DNA was methylated using the EZ DNA Methylation Gold kit (Zymo Research, Irvine, California, USA) as indicated by the manufacturer. CpG islands in the OCT-4 promoter were amplified using the primers presented in Table S2 in Additional file 1[31]. PCR products were then purified using the ExoSAP-IT (Affymetrix, Santa Clara, California, USA) and sequenced. Semi-quantitative analyses were performed using the BiQ Analyzer (Max Planck Institute for Informatics, Saarbrücken, Germany) and BioEdit software (Ibis Biosciences, Carlsbad, California, USA), measuring the relative intensities of the curves from each CpG in the electropherogram. For a comprehensive analysis, we built an intensity graph where the 100% methylated CpG islands are depicted in red and the 100% demethylated islands are depicted in blue. All values in between are expressed by grading the intensity of the corresponding color.

### Statistical analysis

We analyzed continuous variables using the Student *t* test. The significance level was set at 0.05. Data are expressed as mean ± standard deviation.

## Results

**Figure 1:**
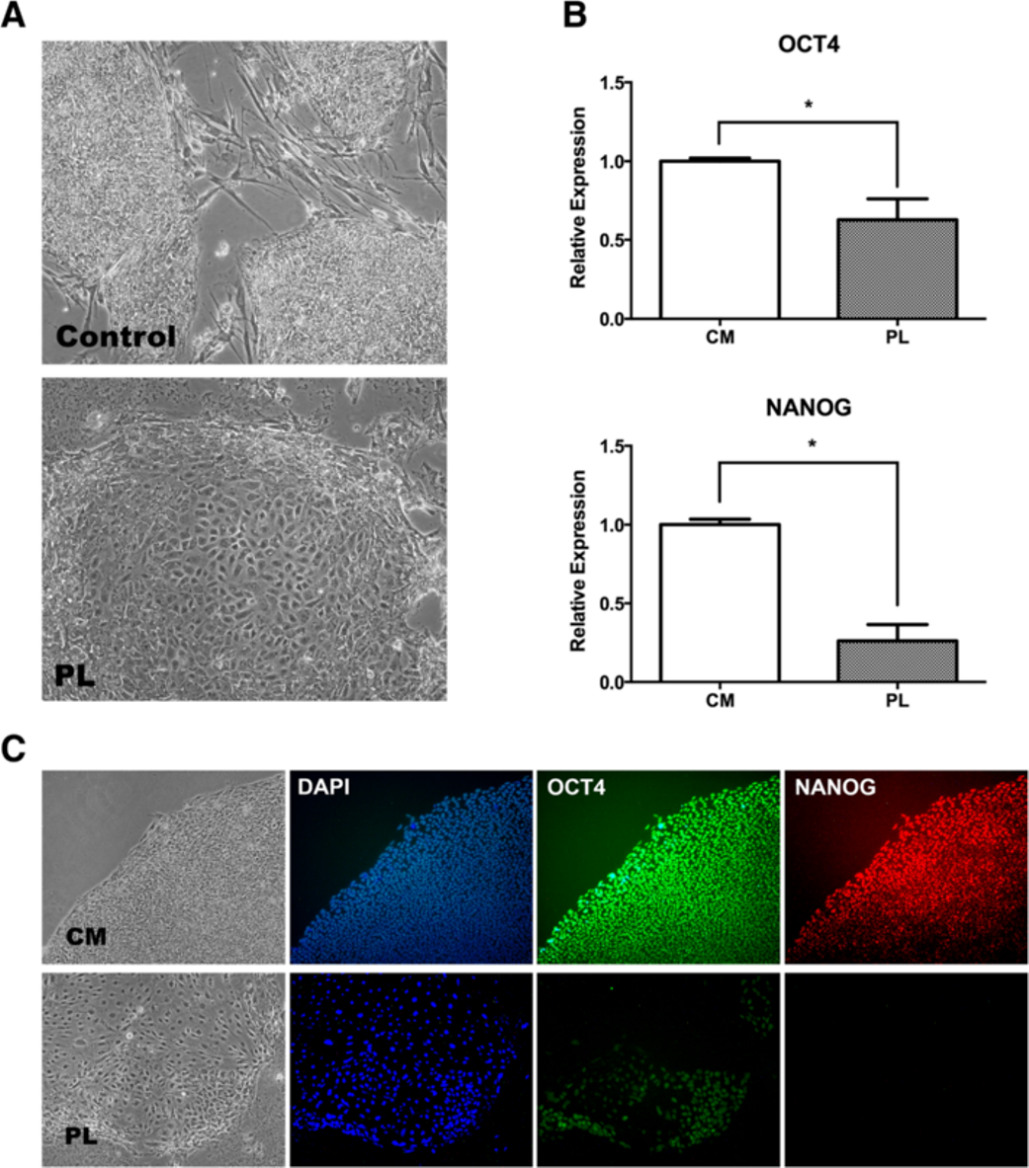
**Use of platelet lysate prompts pluripotent stem cells towards differentiation. (A)** Optical microscopy of pluripotent stem cells (PSC) cultured during 4 days in either standard growth condition (control) or platelet lysate-supplemented medium (PL). Cells gain cytoplasm and differentiation zones are observed. **(B)** mRNA levels of OCT-4 and NANOG in PSC cultured during 4 days in control (CM) or PL, assessed by quantitative PCR. A rapid decline in pluripotent transcription factors is seen (*P <0.05). **(C)** Fluorescence microscopy of 4′,6-diamidino-2-phenylindole (DAPI), OCT-4 and NANOG in PSC cultured as mentioned above. A faint stain for OCT-4 is observed, whereas no expression of NANOG is found.

PL has been shown to include a significant amount of growth factors [6, 7, 32, 33]. We first tested whether these signals would induce differentiation of PSC. The PSC were grown on either inactivated mouse embryonic fibroblasts or Matrigel-coated plates, and Knock-Out Serum Replacement was replaced by PL 10% without basic fibroblast growth factor. This experiment resulted in a rapid differentiation of the pluripotent colonies. As seen in Figure 1, colonies adopted a clear differentiated morphology after 4 days, with irregular shapes of the colonies and cells with increased cytoplasm. We then measured the expression of PSC transcription factors by quantitative RT-PCR, including OCT-4 and NANOG, and found that they significantly declined after 4 days. Finally, we stained PSC colonies for these same pluripotent markers and we found that their expression was lowered or mostly absent by this time. We believe that the significant amount of growth factors induce a rapid differentiation of the PSC, even though we cannot discard the possibility that differentiation towards mesenchymal cells is mainly driven by the lack of pluripotency-supporting conditions.

**Figure 2:**
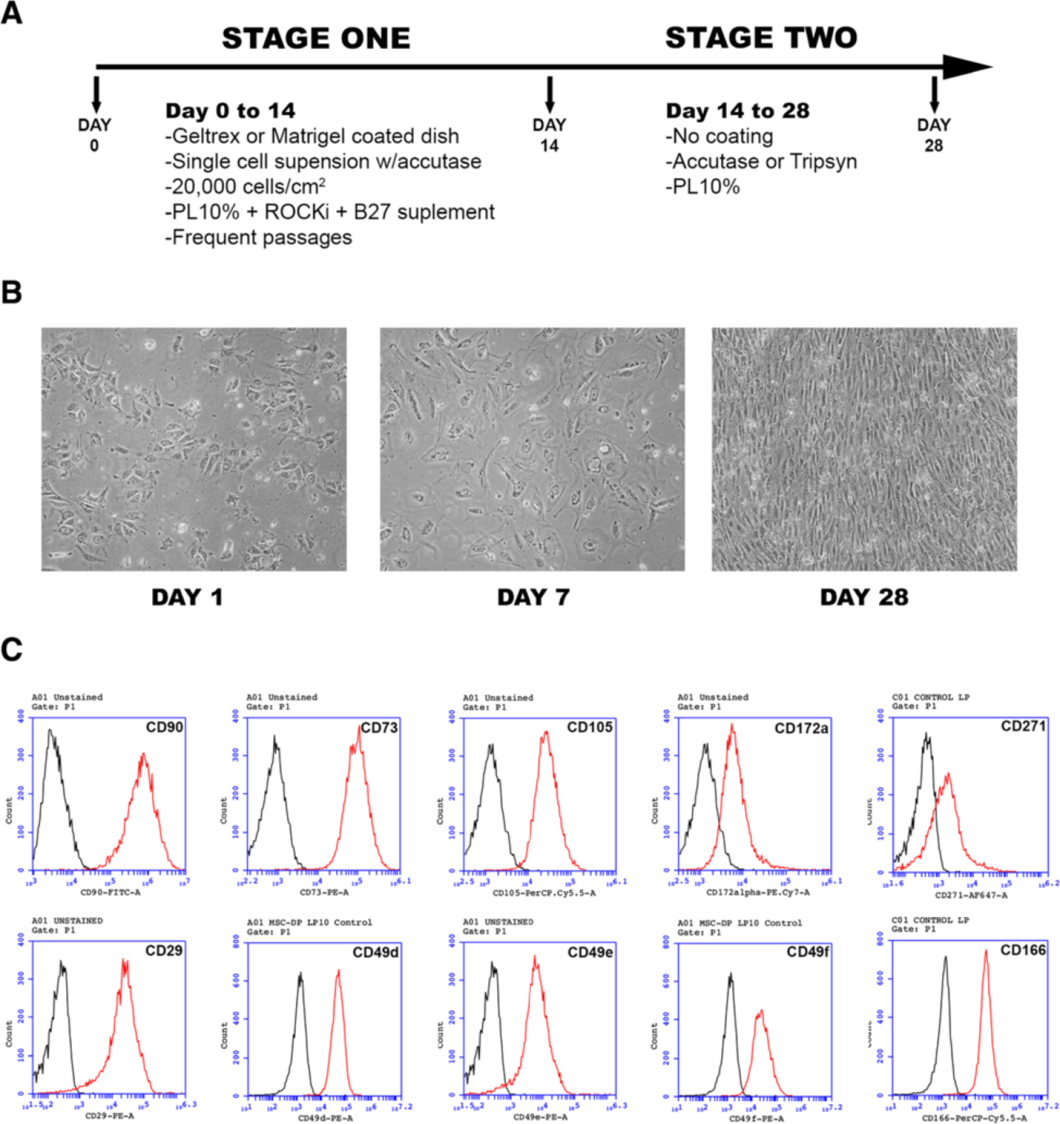
**Use of platelet lysate differentiates pluripotent stem cells towards a mesenchymal phenotype. (A)** Scheme of the differentiation protocol used in this work. **(B)** Microscopic view of the cell’s phenotype progression during protocol. Cells progressively gain cytoplasm and separate from each other. **(C)** After protocol completion, cells are positively stained for mesenchymal stem cell surface markers, analyzed by flow cytometry. Red, cells stained for the indicated marker; black, unstained control. PL, platelet lysate.

We then designed a protocol for differentiating PSC into MSC using PL as cell culture media supplement. After testing several conditions (results not shown), we designed a two-stage protocol (Figure 2A). In the first part, cells transition from the pluripotent stem state to a mesenchymal lineage. During this transition, these cells still require some signals to provide the suitable support to survive. For example, at this stage it is necessary to use a ROCK inhibitor (we use Y-27632 at a concentration of 10 μM) to avoid a massive cell death after plating. We noted that cells were very sensitive at this stage to enzyme dissociation. We then use Accutase for approximately 7 to 10 minutes, enough to dissociate cells to a single cell level [34]. We also observed that a cell surface coating is required. We use Matrigel (diluted 1/40 to 1/60) or Geltrex (diluted 1/100) with similar results. We also tried gelatin-coated plates, but this was ineffective. It is also possible to coat the plate with a gelatin formed with PL resuspended in DMEM, without adding heparin. In approximately 1 hour a light gelatin is formed, which can be easily aspirated leaving a coating at the bottom. We found this coating a good alternative for the first stage of the protocol. Finally, we also add the supplement B27 in this first stage, diluted 1/100. During this stage, culture media was changed every other day and cells were passaged when reached approximately 70 to 80% confluence (usually, three to four times in this period). After approximately 14 days, cells have already transitioned to a mesenchymal state, and the cells become less stringent with the cell culture conditions. From this time point we supplement the medium only with PL 10%, and cells can be passed in a regular way over noncoated plates.

As soon as 24 hours after starting the protocol the cell morphology rapidly changed (Figure 2B), gaining cytoplasm and adopting a rounded form, although they remained attached in small colonies. After a few days these cells begin to separate, and after a few weeks they finally changed to a fusiform shape by 3 to 4 weeks. During this time, the cells continued to grow at a fast rate, and it was necessary to pass them frequently. We analyzed the PD-MSC_PL_ after 30 days of differentiation (and up to three passages later) for several MSC surface markers. These cells strongly expressed the classical MSC markers CD90, CD73 and CD105. They also stained positive for other known MSC markers, including CD166, CD172alpha, and CD271 and integrins (CD29, CD49a, CD49d, CD49e, CD49f and CD51/61). We also found that these cells are negative for several cell surface markers, including CD11b, CD14, CD19, CD34, CD45, CD79a, CD309 and HLA-DR (data not shown). These cells therefore expressed those MSC surface markers that are usually found in MSC isolated from other sources. Finally, we also differentiated PD-MSC using FBS as supplement instead of PL (PD-MSC_FBS_); these cells expressed similar surface markers to PD-MSC_PL_ after 3 to 4 weeks.

**Figure 3:**
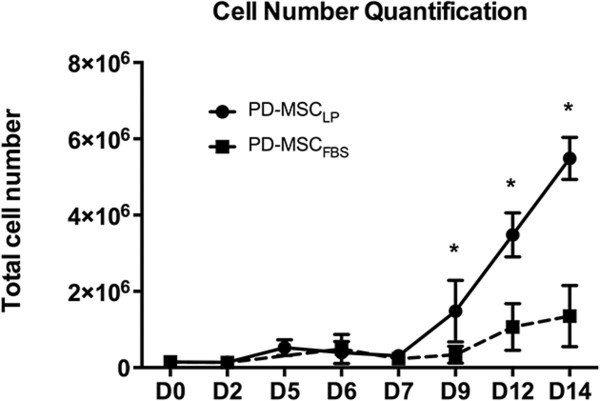
**Platelet lysate supplement increases the yield of mesenchymal stem cells derived.** Initially, 1.5 × 10^5^ pluripotent stem cells were plated and cultured in medium supplemented either with 10% fetal bovine serum (FBS) or 10% platelet lysate (PL). At the indicated days, cells were passed and counted in a flow cytometer. From day nine onwards, PL supplement generates substantially more cells than FBS (*P <0.05). PD-MSC_FBS_, pluripotent-derived mesenchymal stem cells supplemented with fetal bovine serum; PD-MSC_PL_, pluripotent-derived mesenchymal stem cells supplemented with platelet lysate.

**Figure 4:**
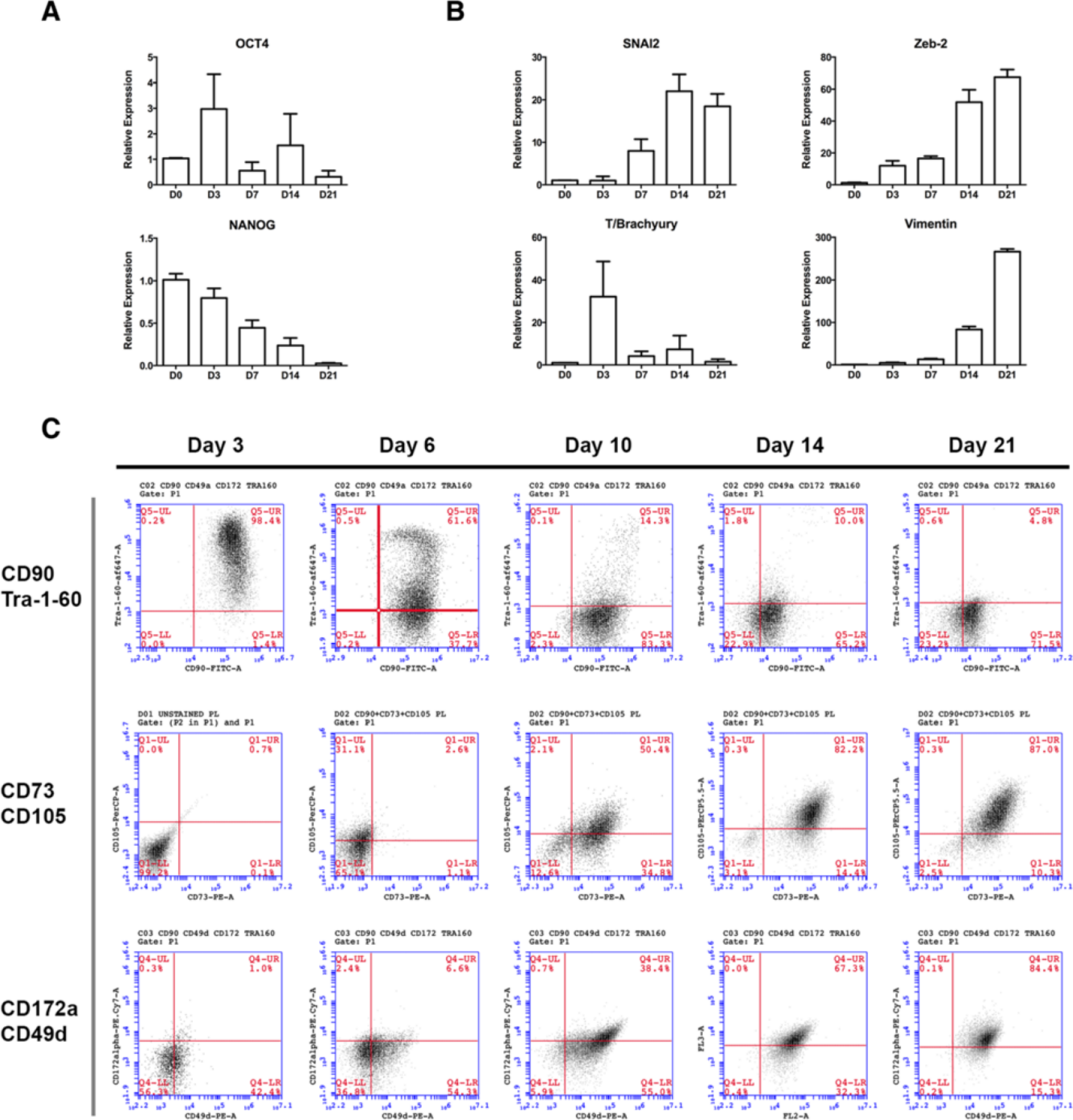
**Kinetics of the epithelial-to-mesenchymal transition during mesenchymal stem cell derivation. (A)** mRNA levels of the pluripotent markers OCT-4 and NANOG along the PD-MSC_PL_ derivation protocol, assessed by quantitative PCR. **(B)** mRNA levels of the epithelial-to-mesenchymal transition markers Snai-2 and Zeb-2, the mesoderm marker T/Brachyury, and the mesenchymal marker Vimentin along the mesenchymal stem cell (MSC) derivation protocol, assessed by quantitative PCR. **(C)** Evolution of CD90/TRA-1-60, CD73/CD105 and CD172a/CD49d during the PD-MSC_PL_ derivation protocol, assayed by flow cytometry. These data point to a slow transformation of pluripotent stem cells into MSC, taking approximately 2 to 3 weeks to complete. PD-MSC_PL_, pluripotent-derived mesenchymal stem cells supplemented with platelet lysate.

In our experiments we noticed that all of the differentiation process takes approximately 3 to 4 weeks to complete. Interestingly, the growth rate with PL supplement was very high (Figure 3), and was considerably higher than that for similar cells derived with FBS. When both supplements were compared, PL was able to produce approximately as many as 25 cells per each PSC after 2 weeks in culture; this number is usually approximately 10 cells when FBS was used as supplement. We then temporarily analyzed the differentiation process looking for a specific point where the cells mature to a PD-MSC_PL_ population. We first analyzed PSC markers by quantitative RT-PCR (Figure 4A). We found that these markers gradually disappeared, and by day 21 the expression was insignificant. We also assessed for markers of the epithelial-to-mesenchymal transition, as this process must necessarily occur. SNAI2 and ZEB2, two transcription factors critical for epithelial-to-mensenchymal transition, significantly increased after a few days of starting the protocol. T/Brachyury was also significantly elevated, but gradually disappeared after a week, as it usually occurs in mesoderm differentiation. Finally, the mesenchymal marker vimentin was found significantly increased at later stages of the differentiation.

We also analyzed the temporal evolution of the mesenchymal differentiation by flow cytometry (Figure 4B). We built several panels of cell surface markers. First, we assessed a two-color panel including MSC marker CD90 (which is also highly expressed in PSC) and Tra-1-60, a specific PSC marker. We found that Tra-1-60 gradually disappeared, and by day 10 was absent. CD90 expression was also slightly reduced after 3 weeks of differentiation, but remained significantly expressed during all of the differentiation time. We then analyzed a three-color panel, gating at CD90^+^cells, and staining for CD73 and CD105, both classical markers of MSC. We found a steady increase in the expression of both markers. However, it was not until day 21 that most cells were CD90^+^CD73^+^CD105^+^. Finally, we built two other three-color panels (CD90/CD172alpha/CD49d and CD90/CD172alpha/CD49a; data not shown). Again, cells become triple positive approximately by day 21 for these markers. In summary, based on the cell culture requirements, the gene expression pattern, and temporal expression of cell surface markers, we conclude that it takes from 14 to 21 days at least for the PSC to differentiate into PD-MSC_PL_. We then considered fully differentiated PD-MSC_PL_ with those cells obtained after 28 days of differentiation, and used them for characterization in the next experiments.

**Figure 5:**
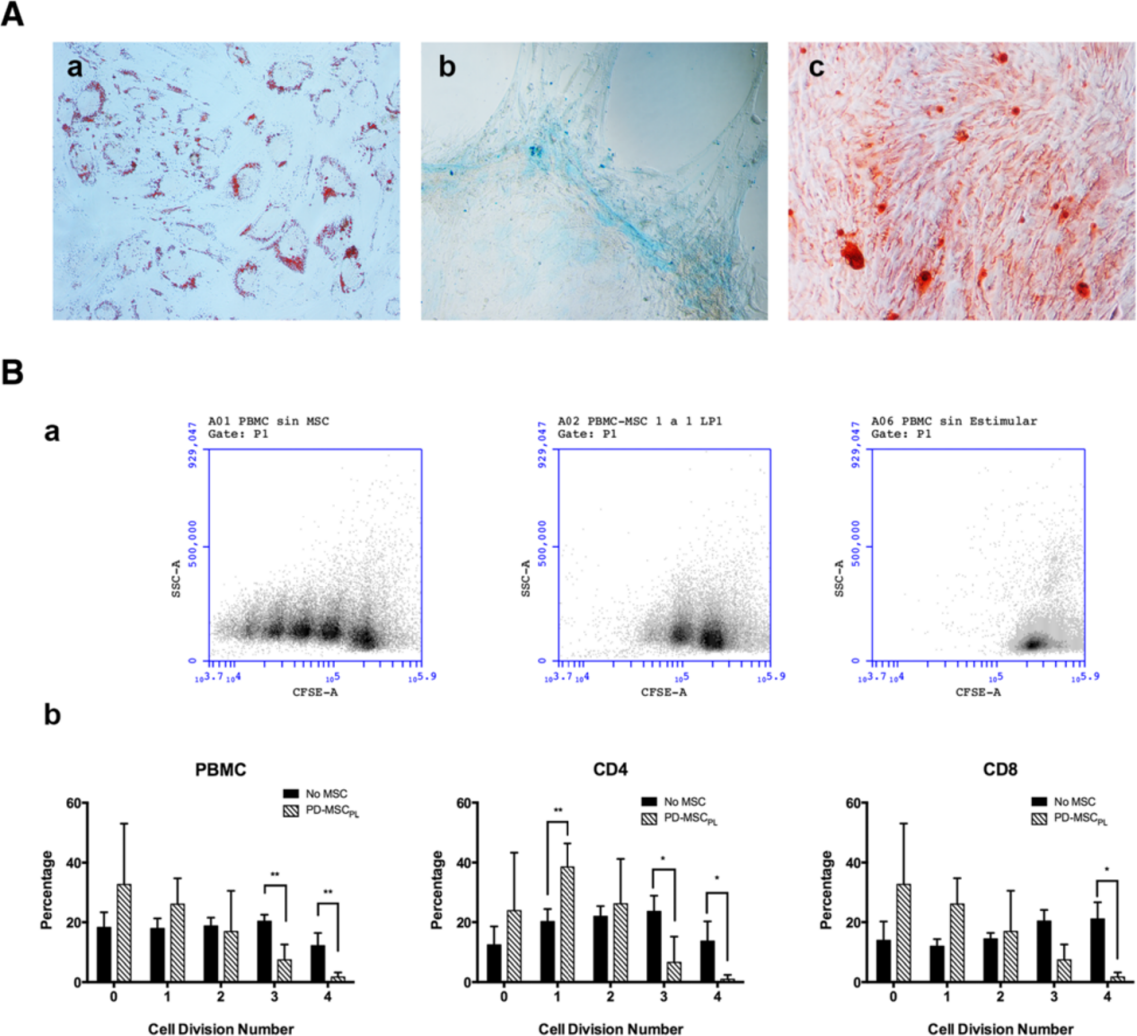
**Pluripotent-derived mesenchymal stem cells supplemented with platelet lysate are fully functional. (A)** Optical microcopy of PD-MSC_PL_ differentiated to an adipogenic (a), chondrogenic (b) or osteogenic (c) lineage. **(B)** Immunomodulation assay. Stimulated peripheral blood mononuclear cells (PBMC) stained with carboxyfluorescein succinimidyl ester (CFSE) show normal proliferation in the absence of mesenchymal stem cells (MSC) (a, left panel) but a reduced proliferation of activated PBMC when cocultured with PD-MSC_PL_ (a, middle panel) compared with that of nonactivated PBMC (a, right panel) in a representative experiment. When stained for CD4 and CD8 lymphocyte populations, a similar finding is seen. Statistical analysis shows significant difference in proliferation from the third doubling onwards (**P <0.1; *P <0.05). PD-MSC_PL_, pluripotent-derived mesenchymal stem cells supplemented with platelet lysate.

We then analyzed the functional properties of the PD-MSC_PL_. We first tested whether PD-MSC_PL_ present multipotent properties. As can be observed in Figure 5A, these cells differentiate into chondroblasts, osteoblasts and adipocytes. After 2 to 3 weeks in the corresponding differentiating media, the cells stained positive for each tissue. We then performed a lymphocyte proliferation assay (Figure 5B) and found that PD-MSC_PL_ present *in vitro* immunomodulating ability. After 4 days in co-culture with PD-MSC_PL_, there was a significant reduction in lymphocyte proliferation. As expected, PD-MSC_FBS_ also present similar immunomodulating properties (data not shown). We further extend our findings by analyzing the subset population of CD4^+^ and CD8^+^ lymphocytes and found that PD-MSC_PL_ are able to significantly reduce the proliferation of both cell types.

**Figure 6:**
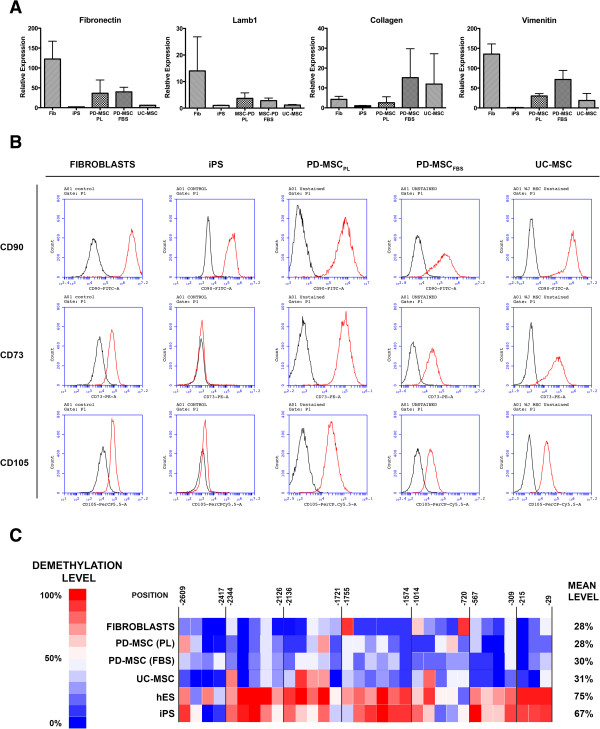
**Pluripotent-derived mesenchymal stem cells supplemented with platelet lysate are similar to other mesenchymal stem cells and different to pluripotent stem cells. (A)** mRNA levels of extracellular matrix components in human foreskin fibroblasts (fib), induced pluripotent stem cells (iPS), PD-MSC_PL_, PD-MSC_FBS_ and umbilical cord mesenchymal stem cells (UC-MSC). **(B)** Expression of mesenchymal stem cell markers in the indicated cell lines. Red, stained cells; black, unstained control. **(C)** Demethylation levels in all cell lines. Each square represents a CpG. Numbers indicate base positions relative to the transcription start site and delimit a CpG island. Globally, the OCT-4 promoter region was found demethylated in human embryonic stem cells (hES) and iPS, whereas all of the mesenchymal stem cells and fibroblasts showed it mostly methylated. FBS, fetal bovine serum; PD-MSC_FBS_, pluripotent-derived mesenchymal stem cells supplemented with fetal bovine serum; PD-MSC_PL_, pluripotent-derived mesenchymal stem cells supplemented with platelet lysate; PL, platelet lysate.

We finally compared the identity of the PD-MSC differentiated using this protocol with other related cells. For this purpose, we compared the properties of PD-MSC_PL_ with fibroblasts, PD-MSC_FBS_, UC-MSC and PSC. The pluripotent stem cell FN2.1 was used as baseline. We first assessed the expression of several markers related to mesenchymal cells, particularly the expression of extracellular matrix proteins. By quantitative RT-PCR we measured the expression of collagen (COL4A1), fibronectin, laminin (the subunit lamb1) and vimentin. Although there was some variation in the expression, we can conclude that PD-MSC_PL_ resemble UC-MSC more than they resemble fibroblasts, and are different to PSC, as expected (Figure 6A). We then analyzed the expression of surface markers in each of these populations (Figure 6B). Most MSC markers are shared between these populations, including fibroblasts, and we could not find any specific surface marker that could distinguish PD-MSC_PL_ from UC-MSC. We could indeed identify some markers that varied from the mesenchymal state to the pluripotent state, and *vice versa*. These markers include the MSC markers and PSC markers. We also performed panel of integrin expressions in these cells (Figure 6; Additional file 2). The integrin expression profile of PD-MSC_PL_ is similar across the mesenchymal cells, but some differences are found with PSC. A profile of the cell surface markers is summarized in the Table 1. Finally, it has been described that the methylation state of the promoter region of the pluripotent gene OCT-4 switches from fibroblast to iPS; also, the OCT-4 promoter is partially methylated in UC-MSC [35–37]. We then assessed all our cell populations for the demethylation state of the promoter of OCT-4 (Figure 6C). We found that this region is mostly demethylated in PSC, as expected in those cells where OCT-4 is highly expressed. The average level of demethylation was similar for both hES and iPS. However, PD-MSC_PL_ presented a mostly methylated state of the promoter of OCT-4. The degree of demethylation was similar to all other MSC, and particularly no different to fibroblasts. Again, these findings support that PD-MSC_PL_ are similar to other MSC, and to some extent similar to fibroblasts.

**Table 1:** **Comparative cell surface expression pattern of the different mesenchymal stem cells analyzed, fibroblasts and pluripotent stem cells**

		Fibroblasts	PSC	PD-MSC _PL_	PD-MSC _FBS_	UC-MSC
Mesenchymal stem cell markers	CD90	+++	+++	+++	+++	+++
	CD73	++	–	+++	+++	+++
	CD105	++	–	+++	+++	+++
	CD166	+++	–	+++	+++	+++
	CD172alpha	+	–	+	+	++
	CD271	–	+	+	+	+
Pluripotent stem cell markers	Tra-1-60	–	+++	–	–	–
	CD133	–	+++	–	–	–
	CD309	–	++	–	–	–
Integrins	CD29	+++	+++	+++	+++	+++
	CD49a	+++	–	+++	+	++
	CD49d	+++	–	+++	+++	+++
	CD49e	+++	+++	+++	+++	+++
	CD49f	+++	+++	+++	+++	+++
	CD51/61	+++	–	+++	+++	+++

Markers are grouped according to phenotype. No major difference was found between fibroblasts, PD-MSC_PL_, PD-MSC_FBS_ and UC-MSC. Some markers, however, are useful to distinguish between PSC and mesenchymal cells. –, no expression; +, mild expression; ++, medium expression; +++, strong expression. PD-MSC_FBS_, pluripotent-derived mesenchymal stem cells supplemented with fetal bovine serum; PD-MSC_PL_, pluripotent-derived mesenchymal stem cells supplemented with platelet lysate; PSC, pluripotent stem cells; UC-MSC, umbilical cord mesenchymal stem cells.

## Discussion

We present in this paper a detailed protocol to derive MSC from PSC. Our protocol involves simple steps, and generates a significant amount of cells in approximately 1 month. The main feature of this new protocol is the use of a therapy-grade supplement, PL. This supplement is easy to produce in great quantities, and it allows a significant expansion of the mesenchymal cells. We produced and tested several PL batches, and there seems to be no differences in the final product in terms of the ability to generate PD-MSC_PL_, although this should be formally tested. However, other papers analyzed the differences in batches and found that they all present similar results [38, 39]. We also tested several hES and iPS cell lines, with similar results in all of them. We therefore conclude that the method is reliable enough to be applied in different settings.

PD-MSC are somehow intriguing. Since their description in 2005, many investigators have characterized a population of cells outgrowing from PSC that shared many features of MSC. Moreover, an early publication on the culture of PSC over different matrixes described the outgrowing of fibroblastoid cells, which gave nutritional support to PSC [40–42]. These cells were probably PD-MSC, although they were not characterized as MSC should be. We then hypothesized that it is an intrinsic feature of PSC to differentiate into a mesenchymal state whenever adverse conditions for staying as PSC are found. We did not address this hypothesis, although an interesting observation supporting it is that we do not see a significant apoptotic rate while the proper culture conditions are met, and hence most if not all cells that undergo differentiation end up in a mesenchymal state. As opposed to embryoid body generation where the three germinal layers are generated, this procedure – which shares the unspecificity of the wide combination of growth factors that FBS or PL contains – yields only mesenchymal cells. It remains to be determined what feature of this process drives the differentiation to mesenchymal cells (that is, two-dimensional vs. three-dimensional culture conditions).

PL has been used for many years as a medium supplement. PL became popular a few years ago for growing MSC due to its effectiveness and low cost. PL has shown some advantages compared with FBS, such as the greater growing rate when MSC are grown with PL. We also found a similar result, but in our case we found that the PL supplement significantly increased the number of differentiated cells. Regarding costs, PL is discarded once platelet units expire, which is usually after 5 days of isolation. Many biocompatible mediums are based on defined components, most of them expensive, particularly when considered for a significant scale up in cell production. With PL, a single batch of 10 units would produce an amount of supplement enough for 5 to 10 l of medium. Scaling up then would not be problematic. A potential disadvantage of PL, however, is the potential biological risk. Platelet units may contain virus (that is, HIV, cytomegalovirus, and so forth) or parasites (that is, *Tripanozoma cruzii* in our country), which may be transmitted to a potential recipient if cells are then used in clinical treatments. Although the risk is minimal after the current protocols for blood testing are properly applied, the risk is not zero. However, it is currently unclear whether this risk surpasses the risk of using FBS, considering potential problems of using an animal-derived product in humans. In any case, blood derivate transfusion is broadly expanded without major problems with transmissible diseases, and PL supplement has been applied in clinical trials using MSC. Recently, Frobel and colleagues analyzed the epigenetic changes during redifferentiation of PSC towards MSC using PL [43]. Notably, they found that PD-MSC seem to provide a more standardized source of MSC due to epigenetic resetting during cell reprogramming, but they proposed that these cells have to be more thoroughly assessed.

We found significant immunomodulatory properties of these cells. We performed a lymphocyte proliferation test where PD-MSC_PL_ were found to significantly reduce the immune activation. Even though this is a common feature in all MSC, we performed a test with prestimulated lymphocyte. A previous publication found that UC-MSC were not able to reduce the immune response when lymphocytes were prestimulated, as in our experiment [44]. The reason why we prestimulated the lymphocyte was that we found concanavalin-A was toxic for PD-MSC_PL_. It remains to be determined whether this is a particular effect in PD-MSC_PL_, since concanavalin-A toxicity has not been reported with other MSC. In any case, our preliminary test confirms that UC-MSC are not able to immunomodulate prestimulated lymphocytes. The case for a significant immunomodulation of PD-MSC has also been found in other papers. Recently, Kimbrel and colleagues and Wang and colleagues reported that PD-MSC are very potent immunomodulators, and are even able to improve outcomes in a sclerosis multiple mouse model, which is typically resistant to MSC from other sources [26, 45]. These findings support previous reports where PD-MSC where found to be effective in modulating the lymphocyte proliferation, but also to regulate the natural killer cell response, an effect not observed with bone marrow-derived MSC [24, 25]. In summary, our experiments support the concept that PD-MSC are at least as potent as other MSC for immunomodulation.

## Conclusions

We describe an easy, low-cost, therapy-grade protocol to generate a significant number of PD-MSC. We believe this protocol could be applied in therapies in which autologous iPS are generated and then large numbers of MSC are produced, and this process could be preferable instead of multiple bone marrow aspirations or fat biopsies.

## Electronic supplementary material

Additional file 1: Table S1.: Presenting the primers used for quantitative PCR analysis and Table S2 presenting the primers used for demethylation analysis in the OCT4 promoter region. (DOC 524 KB)

Additional file 2: Figure S1: Showing the complete panel of all markers assayed in human foreskin fibroblasts (fibroblasts), PSC (iPS), PD-MSC_PL_, PD-MSC_FBS_ and UC-MSC. Red, stained cells; black, unstained control. (ZIP 2 MB)

## Abbreviations

BSA: bovine serum albumin
DMEM: Dulbecco’s modified Eagle’s medium
FBS: fetal bovine serum
hES: human embryonic stem cells
iPS: induced pluripotent stem cells
MSC: mesenchymal stem cells
PBMC: peripheral blood mononuclear cells
PBS: phosphate-buffered saline
PD-MSC: pluripotent-derived mesenchymal stem cells
PD-MSC_FBS_: pluripotent-derived mesenchymal stem cells supplemented with fetal bovine serum
PD-MSC_PL_: pluripotent-derived mesenchymal stem cells supplemented with platelet lysate
PL: platelet lysate
PSC: pluripotent stem cells
UC-MSC: umbilical cord mesenchymal stem cells.
