# Supplementary material for: A therapy-grade protocol for differentiation of pluripotent stem cells into mesenchymal stem cells using platelet lysate as supplement
Source: Stem Cell Res Ther. 2015 Jan 12;6(1):6. doi: 10.1186/scrt540 (PMC4417240; doi:10.1186/scrt540)
Supplement: Supplementary file 1 — Additional file 1: Table S1.: Presenting the primers used for quantitative PCR analysis and Table S2 presenting the primers used for demethylation analysis in the OCT4 promoter region. (DOC 524 KB) [file 13287_2014_422_MOESM1_ESM.doc]

| Table 1 (supplementary): Primers used for qPCR analysis. | | |
| --- | --- | --- |
| Gene | Forward Primer | Reverse Primer |
| OCT4 | GCTGGAGAAGGAGAAGCTGG | GCTAAGCTGCAGAGCCTCAA |
| NANOG | AAAGAATCTTCACCTATGC | GAAGGAAGAGGAGAGACA |
| IDO | AGAGTCAAATCCCTCAGTCC | AAATCAGTGCCTCCAGTTCC |
| LAMC1 | GGCCCAGGATGTCAAAGATG | TGTTCTCTACATGGGCACG |
| Fibronectin | AAGACCAGCAGAGGCATAAGG | CACTCATCTCCAACGGCATAATG |
| Colagen | GCCAAGTATCTCACCTGGATCA | GGACTACCTGGAACAAAAGGG |
| Vitronectin | GAGTGCAAGCCCCAAGTGAC | GCCATCGTCATAGACCGTGT |
|  |  |  |

| Table 2 (supplementary): Primers used for demethylation analysis in the OCT4 promoter region. | | |
| --- | --- | --- |
| OCT4 Promoter Region  (relative to transcription start site) | Forward Primer | Reverse Primer |
| -2609 to -2417 | TTAGGAAAATGGGTAGTAGGGATTT | TACCCAAAAAACAAATAAATTATAAAACCT |
| -2344 to -2126 | ATTTGTTTTTTGGGTAGTTAAAGGT | CCAACTATCTTCATCTTAATAACATCC |
| -2136 to -1721 | GGATGTTATTAAGATGAAGATAGTTGG | CCTAAACTCCCCTTCAAAATCTATT |
| -1755 to -1574 | AATAGATTTTGAAGGGGAGTTTAGG | TTCCTCCTTCCTCTAAAAAACTCA |
| -1014 to -720 | GAAGGGGAAGTAGGGATTAATTTT | CAACAACCATAAACACAATAACCAA |
| -567 to -309 | TAGTTGGGATGTGTAGAGTTTGAGA | TAAACCAAAACAATCCTTCTACTCC |
| -215 to -29 | AAGTTTTTGTGGGGGATTTGTAT | CCACCCACTAACCTTAACCTCTA |
