# Supplementary figures and images for: A therapy-grade protocol for differentiation of pluripotent stem cells into mesenchymal stem cells using platelet lysate as supplement
Source: Stem Cell Res Ther. 2015 Jan 12;6(1):6. doi: 10.1186/scrt540 (PMC4417240; doi:10.1186/scrt540)

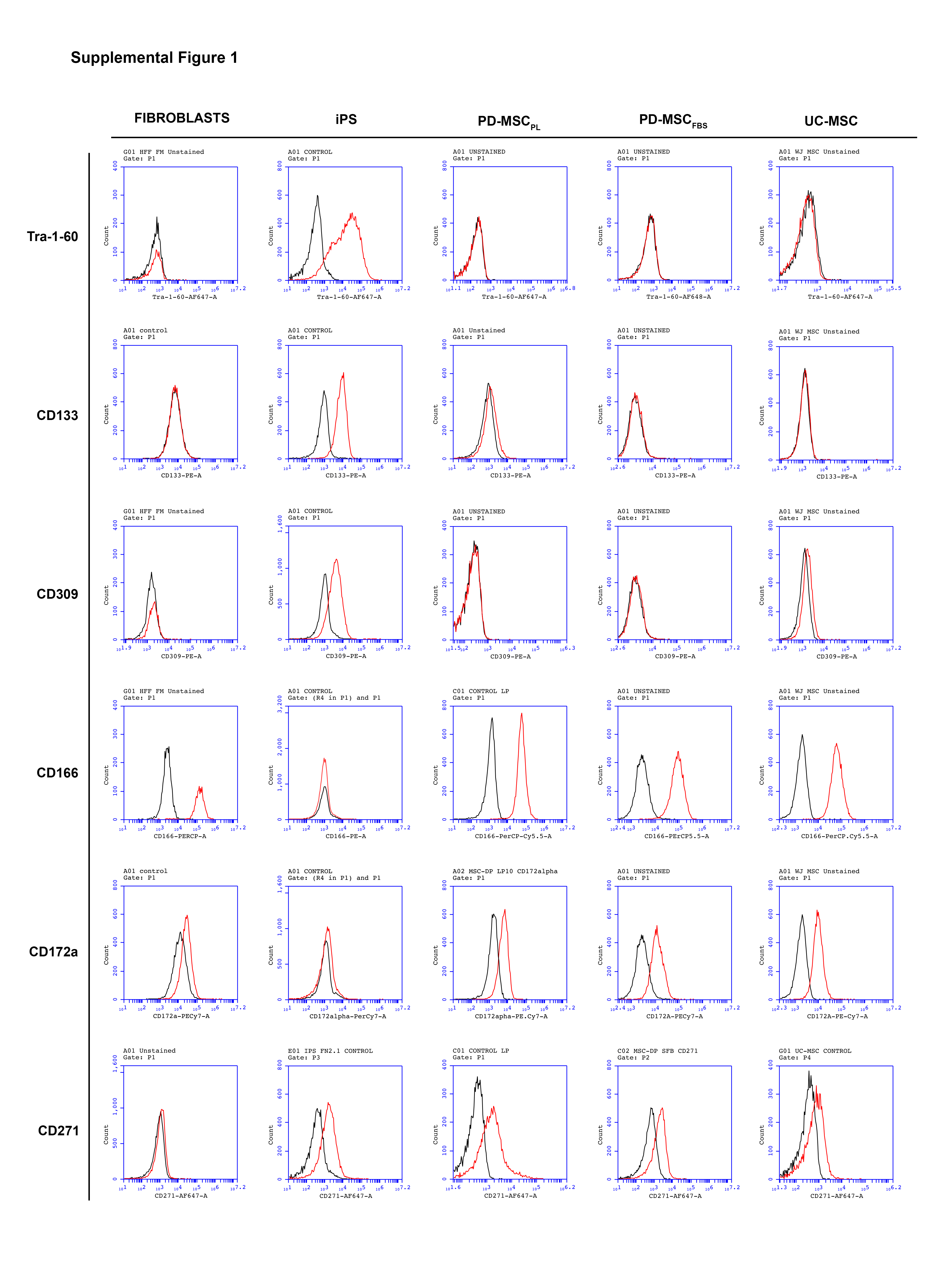

Supplement: Supplementary file 2 — Additional file 2: Figure S1: Showing the complete panel of all markers assayed in human foreskin fibroblasts (fibroblasts), PSC (iPS), PD-MSCPL, PD-MSCFBS and UC-MSC. Red, stained cells; black, unstained control. (ZIP 2 MB) [file 13287_2014_422_MOESM2_ESM.zip › 1531762861442432_add1.tiff]

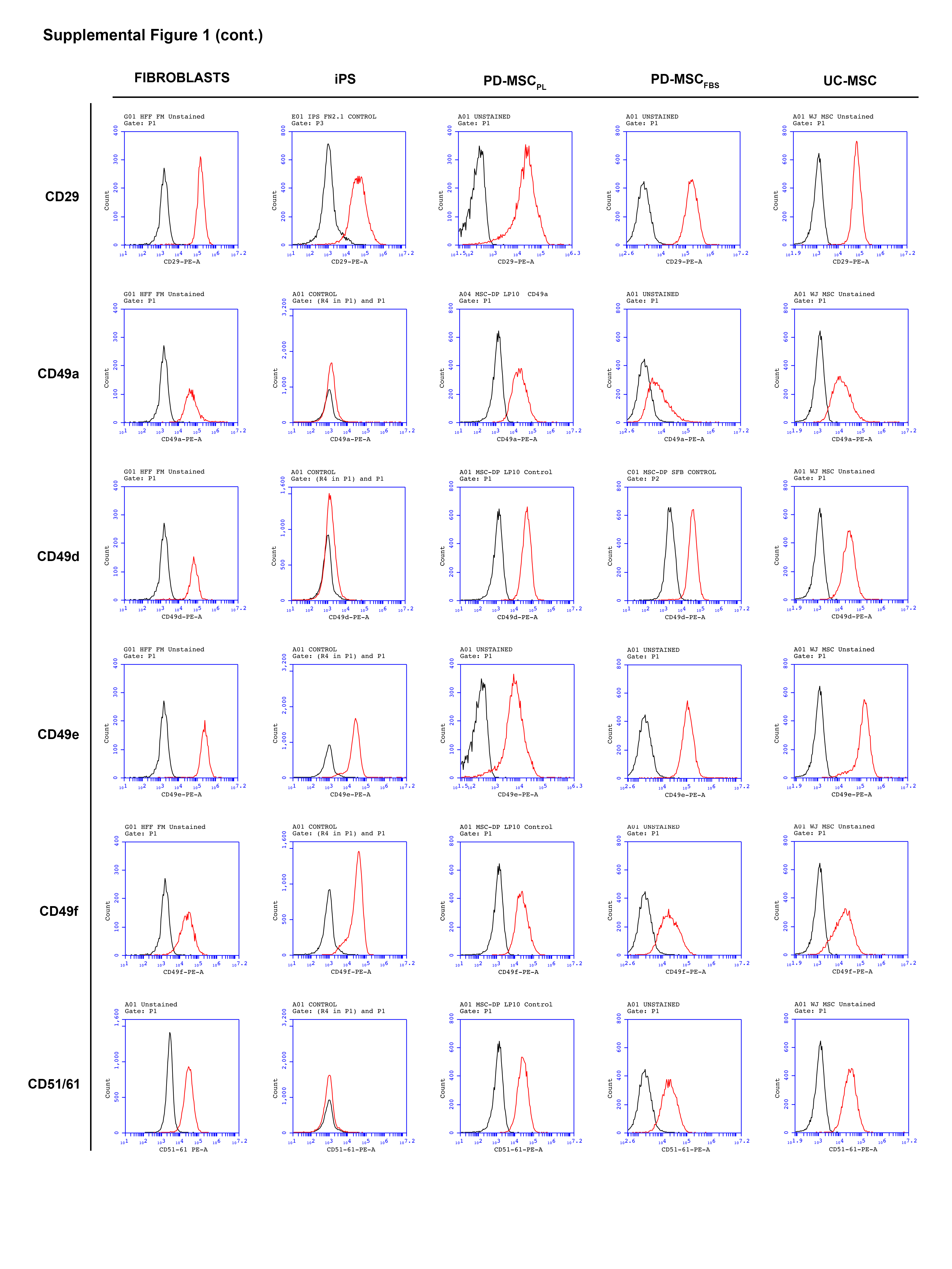

Supplement: Supplementary file 2 — Additional file 2: Figure S1: Showing the complete panel of all markers assayed in human foreskin fibroblasts (fibroblasts), PSC (iPS), PD-MSCPL, PD-MSCFBS and UC-MSC. Red, stained cells; black, unstained control. (ZIP 2 MB) [file 13287_2014_422_MOESM2_ESM.zip › 1531762861442432_add2.tiff]
